# Mercuric-sulphide based metallopharmaceutical formulation as an alternative therapeutic to combat viral and multidrug-resistant (MDR) bacterial infections

**DOI:** 10.1038/s41598-023-43103-z

**Published:** 2023-10-04

**Authors:** Kootharasan Malarvizhi, Durai Ramyadevi, B. Narayanan Vedha Hari, Hema Bhagavathi Sarveswari, Adline Princy Solomon, H. Fang, R. H. Luo, Y. T. Zheng

**Affiliations:** 1 Pharmaceutical Technology Laboratory (#214, ASK-II), School of Chemical and Biotechnology, SASTRA Deemed University, Thanjavur, 613401 India; 2 Centre of Molecular and Macromolecular Studies, Polish Academy of Sciences, Sienkiewicza 112, 90-363 Lodz, Poland; 3 Quorum Sensing Laboratory, Centre for Research in Infectious Diseases, School of Chemical and Biotechnology, SASTRA Deemed to be University, Thanjavur, 613401 India; 4 Kunming Institute of Zoology, Chinese Academy of Sciences, Kunming, Yunnan China

**Keywords:** Microbiology, Medical research, Nanoscience and technology

## Abstract

According to the Global Antimicrobial Resistance and Use Surveillance System (GLASS) data, antibiotic resistance escalates more challenges in treatment against communicable diseases worldwide. Henceforth, the use of combinational antimicrobial therapy and metal-conjugated phytoconstituents composites are considered as alternatives. The present study explored the efficacy of mercuric-sulfide-based metallopharmaceutical, *Sivanar Amirtham* for anti-bacterial, anti-tuberculosis, anti-HIV therapeutics and toxicity profile by haemolytic assay, first of its kind. The anti-bacterial study was performed against both gram-positive and gram-negative pathogens including *Staphylococcus aureus* (ATCC 29213)*,* Methicillin-resistant *Staphylococcus aureus* (MRSA: ATCC 43300), *Enterococcus faecalis* (ATCC 29212)*, Pseudomonas aeruginosa* (PA14) and *Vibrio cholerae* (MTCC 3905) by agar well diffusion assay, wherein the highest zone of inhibition was identified for MRSA (20.7 mm) and *V. cholerae* (34.3 mm) at 25 mg/mL. Furthermore, the anti-tuberculosis activity experimented by microtitre alamar blue assay against *M. tuberculosis* (ATCC 27294) demonstrated significant activity at the concentration range of 12.5–100 µg/mL. Additionally, the anti-HIV efficacy established by the syncytia inhibition method using C8166 cell lines infected with HIV-1_IIIB_, showed a significant therapeutic effect. The in-vitro toxicity assay proved *Sivanar Amirtham* to be non-haemolytic and haemocompatible. The physicochemical characterization studies revealed the nano-sized particles with different functional groups and the distinctive metal–mineral complex could be attributed to the multi-site targeting ability. The rationale evidence and scientific validation for the efficacy of *Sivanar Amirtham* ensures that it could be proposed as an alternative or adjuvant for both prophylactics and therapeutics to overcome HIV infection and antimicrobial resistance as well as the multi-drug resistance challenges.

## Introduction

Antimicrobials are active pharmaceutical ingredients used for the prevention and treatment of infections, which include antibiotics, antivirals, antifungals, and antiparasitic. In particular, antibiotics are the medications used to treat bacterial infections and play a pivotal role in extending the life span of patients undergoing chemotherapy, surgeries, and chronic treatment for infectious diseases^1^. In developing countries and highly populated areas, these drugs help in reducing the morbidity and mortality rate caused by infections due to poor sanitation resulting in numerous water-borne, food-borne, and air-borne infections^2^. Even though antibiotic resistance occurs naturally, the overuse of antibiotics and inappropriate prescription of antibiotics drive the development of resistance. As per the epidemiological studies reported, antibiotic consumption is directly related to the emergence and dissemination of resistant strains^3^. The mortality rate due to antimicrobial resistance in developed as well as developing countries is estimated to be ten million per year by 2050^4^. In 2010, the highest antibiotic usage of 12.9 billion units was documented in India, and in 2019, about 77.1% of systemic antibiotic sales were witnessed^5^. According to an Indian Council of Medical Research (ICMR) report, the resistance towards broad-spectrum antibiotics had increased every year by 5–10%^6^ and the resistance cases are likely one in six people (fic.nih.gov). The emergence of antibiotic-resistant strains is detected among both gram-positive and gram-negative species including *Staphylococcus aureus, Enterococcus* species, *Pseudomonas aeruginosa, Acinetobacter* species, *Escherichia coli, Klebsiella pneumonia* and *Neisseria gonorrhoeae*^7^*. P. aeruginosa* producing metallo-beta-lactamase enzyme is an important cause of nosocomial infections, which also mediates the resistance to carbapenems and its prevalence is predicted to be in the range of 7–65%. A recent study reported about 31.21% of Methicillin-Resistant *S. aureus* (MRSA) from the analysis of 487 specimens, and Vancomycin-resistant *S. aureus* strains were found to be higher in hospitalized patients, whereas Clindamycin resistance prevailed high in community-acquired-MRSA^8^. Recently, *Enterococcus faecalis* strains have shown resistance against Teicoplanin and Vancomycin^2^. *Mycobacterium tuberculosis* (TB) strains have developed resistance against first-line treatment drugs like Isoniazid or Rifampicin. Due to the resistance developed against anti-TB drugs including Fluoroquinolones and other injectable drugs like Kanamycin and Capreomycin, it is more challenging to treat Extensively Drug-Resistant Tuberculosis^3^. Recent studies in India have shown that the multi-drug resistant strain *Vibrio cholera* isolate ‘O1’ has developed resistance towards Ampicillin, Tetracycline, Ciprofloxacin, Furazolidone, and Ceftriaxone^9,10^.

The strategies required to combat antibiotic resistance include the use of multiple drugs or combinatorial antibiotic therapy, design, and development of new antibiotics, co-administration of non-antibiotic drugs or adjuvants that help in inhibiting antibiotic degradation and resistance, and traditional therapeutic approaches to tackle the complications. The development of new antibiotics involves more time and unreasonable costs of up to a few hundred million dollars^2^. Along with the existing drug resistance pathogens causing tuberculosis, malaria, and cholera, further challenges are imposed by the multi-drug resistant strains of several other infections^11^. Combinational antimicrobial therapy is an effective therapeutic strategy, which induces antimicrobial synergism that will be an alternative to standard monotherapy and also used against multi-drug resistant species^12,13^. Phyto therapeutic agents or polyherbomineral medicines are considered one of the valuable options against antibiotic resistance, especially in India, where the traditional system of medicine is followed, for several decades for treating various infectious diseases. These medicinal products are supposed to work in a similar mechanism to antibiotics by either killing the bacteria or battling its proliferation^14^. Polyherbomineral medicines also termed metallopharmaceuticals, constitutes complex composition of metals and minerals along with herbal ingredients, which possess effective antimicrobial activity due to their ability to target multiple sites such as genetic material, cellular membrane, and reactive oxygen species (ROS)-mediated cellular pleiotropic effects. The nanoparticles composed of metals or metal oxides of silver, copper, gold, cupric oxide, zinc oxide, and titanium dioxide were proven to be potential antibacterial agents by their effective interactions with pathogen membranes^15,16^. Metals have played a notable role in elucidating antimicrobial properties since ancient times till date. Silver is a well-known antimicrobial agent used as a topical ointment for treating wounds. Mercury salts are found to be effective against *Salmonella typhi*,* Staphylococcus aureus*,* Escherichia coli*, and *Bacillus subtilis*^17^. Also, the metal-based complexes are used for other therapeutic purposes viz., Arsphenamine (Salvarsan), an organometallic complex containing arsenic is used for the treatment of syphilis; Mercurochrome, a mercury-based formulation is an antiseptic agent; and Auranofin, a complex of gold is used to treat rheumatoid arthritis^18^.

Siddha and Ayurveda are part of the traditional Indian system of medicines, which embraces various polyherbal and herbomineral formulations possessing noteworthy activity against several microbial pathogens. Ayurvedic formulations like *Hartal Bhasma* (arsenic trisulphide), *Tambra Bhasma* (Copper), *Yashad Bhasma* (Zinc), etc. containing specified minerals or metals are found to exhibit remarkable antimicrobial activity^19,20^. Similarly, Siddha formulations like *Vajrakandi* (cinnabar, calomel, hydrargyrum perchloride), *Sandmarutham* (cinnabar, calomel, sulphur), *Linga Chendooram* (cinnabar), *Mupoora Chendooram* (cinnabar, mercuric subchloride, mercuric sulphide), etc. containing the unique composition of minerals and metals exhibit effective antibacterial properties^21–23^.

It is well-known that TB infection is more likely to be associated with HIV-positive patients as a co-infection, which further increases the severity of the disease and decreases patient immune responses. In fact, it is one of the major causes of the increased mortality rate among HIV patients (by approximately three lakhs)^24,25^. Hence, this notable co-epidemic concern has to be considered and treated appropriately. The combinatorial treatment is affected by the development of multi-drug resistant strains and also due to the complications in prescribing anti-retroviral drugs along with anti-TB agents. So, it is evident that a need for an alternative drug formulation that can overcome these complications together^26^. The analogues with metal centers^27^ are found to be more effective. Therefore, instead of developing new entities, repurposing existing formulations will provide a better resolution owing to the problem. Recently, the anti-malarial agent Chloroquine has been repurposed for anti-HIV treatment^28^. Sulphated carrageenan is reported to be effective against HIV-1 by inhibiting the entry of the virus by directly binding with the glycoprotein 120^29^. Similarly, mercury exhibits significant activity by inhibiting the HIV-1 protease^30^.

With this background, the current study attempted to determine both the antibacterial and antiviral efficacy of the traditional mercuric-sulphide-based metallopharmaceutical formulation, *Sivanar Amirtham*. *Sivanar Amirtham* comprises detoxified mercury, detoxified sulphur, detoxified red orpiment, detoxified borax, detoxified *Dryopteris filix-mas*, detoxified *Aconitum ferox*, *Zingiber officinale, Piper longum, and Piper nigrum* in the determined ratio as described in the standard monograph specifications^31^. Since ancient times, it is recommended in clinical practice to treat various infections, and respiratory diseases including tuberculosis, leukoderma, gastric ulcers, seizures, and piles, and also as an antidote for scorpion bites^32^. However, traditional medicine lacks validated scientific proof-of-evidence to explain their proposed therapeutic activity and understand the mechanisms of action. Therefore, in the present research, *Sivanar Amirtham* was prepared as per the standard traditional protocol and characterized for the physicochemical properties using modern analytical techniques such as field emission scanning electron microscopy (FE-SEM), Fourier transform infra-red (FTIR) spectroscopy, X-ray diffraction (XRD) and X-ray fluorescence spectroscopy (XRF). Finally, the formulation was subjected to in-vitro pharmacological screening for anti-microbial efficacy against *S. aureus* (MRSA and MSSA),* P. aeruginosa, E. faecalis,* and *V. cholera;* anti-tuberculosis activity by Alamar blue assay; anti-HIV activity by syncytia inhibition method against C8166 cell lines infected with HIV_IIIB_; and toxicity profile by haemolytic assay, reported for the first time, to validate and provide scientific evidence for intended therapeutic uses.

## Material and methods

### Materials

The raw materials like *Zingiber officinale, Piper longum, Piper nigrum, Dryopteris filix-mas, Aconitum ferox,* and red orpiment were procured from Rajan & Co., Chennai, Tamil Nadu, India. The herbals used were identified and authenticated by Dr. Ravichandran, Botanist, SASTRA Deemed University, Thanjavur by standard pharmacognosy screening procedures and the herbarium of the same has been archived at the department (Herbarium no: CARISM 00157-00161). The experimentation using plants was performed in accordance with the International Union for Conservation of Nature (IUCN). Moreover, the plants used were not categorized in the IUCN endangered or extinct species list. Sulphur, Mercury, Borax, Mueller-Hinton Agar, Middlebrook 7H9 broth base, and Alamar reagent were obtained from Sigma-Aldrich, Mumbai, and verified for their identity and purity. All the chemicals and reagents used were of analytical grade.

### Preparation of the Mercuric-Sulphide-based metallopharmaceutical *Sivanar Amirtham*

The *Sivanar Amirtham* formulation included metals, minerals, and herbal ingredients, and the preparation process involved two steps. In the first step, the detoxification of the mercury, sulphur, red orpiment, borax, *D. filix-mas,* and *A. ferox* was intended to crucially remove the toxic impurities and convert them into biologically assimilable form as per the standard protocol mentioned in the Siddha formulary of India and the authorized reference texts^33^. The second step involved two sub-stages, wherein the initial phase is the development of *Kajjali*, followed by the second phase to formulate *Sivanar Amirtham* product. *Kajjali* (Mercuric Sulphide), the prime component of the formulation was prepared by trituration of 10 g of detoxified mercury and 10 g of detoxified sulphur initially for two hours in hand kalvam at the rate of 45 cycles/min, followed by the constant grinding for 8 h in the motorized kalvam at the rate of 25 rpm until luster-free mercuric sulphide (β-HgS) powder was obtained. The obtained mercuric sulphide powder (10 g) was further triturated with other ingredients such as detoxified borax, detoxified red orpiment, detoxified *A. ferox*, detoxified *D. filix-mas*, *Z. officinale, and P. longum,* by the subsequent addition of equal parts of individual materials (10 g each) one-by-one in the mentioned order, with intermittent trituration for 4 h after each addition. Finally, 8 parts of *P. nigrum* (80 g) were incorporated into the mixture and continuously triturated using an end-runner at 25 rpm for 10 days and the time accounted for 8 h per day^34^.

### Analytical characterization of Mercuric-sulphide-based Metallopharmaceutical *Sivanar Amirtham*

The powder surface morphology and the elemental composition of the *Sivanar Amirtham* a metallopharmaceutical product were assessed using Field emission scanning electron microscopy coupled with energy dispersive X-ray spectroscopy (SEM, JEOL, JSM-6360, Japan). The sample was analyzed after coating with a thin film of gold about 20 nm using Sputter Coater (Q150R ES, Quorum Technologies, East Sussex, England). The particle size and distribution of the sample were analyzed using a Zeta sizer (Malvern Instruments, UK) through dynamic light scattering technique. To understand the chemical nature of the formulation, Fourier transform infrared (FTIR) spectroscopy analysis was carried out using the KBr pellet technique. The sample was triturated with previously dried and saturated potassium bromide and converted into pellets, which were exposed to an infrared source to record the spectrum in the wavelength range between 4000 and 400 cm^−1^. The crystalline property of the product was assessed from its interference patterns using a powder X-ray diffractometer (D8 Focus, Bruker AXS, Germany). The metallopharmaceutical product was investigated for its elemental composition by X-ray fluorescence spectroscopy (Bruker AXS S8 Tiger, Germany) analysis using a 4 kW intensity X-ray tube with Rhodium anode. The sample was homogenized in mortar and pestle, dried at 45 °C, and then pelletized using a hydraulic press to get homogenized and smooth surfaced pellets for analysis^34^.

### In-vitro anti-microbial activity of Mercuric-sulphide based Metallopharmaceutical *Sivanar Amirtham*

#### Bacterial strains and growth conditions

Standard reference bacterial strains including Gram-positive bacteria *Staphylococcus aureus* (ATCC 29213-MSSA and ATCC 43300-MRSA) and *Enterococcus faecalis* (ATCC 29212), and Gram-negative bacteria *Pseudomonas aeruginosa* (PA14) and *Vibrio cholerae* (MTCC 3905) were used in this study. The ATCC strains and MTCC strains were procured from HiMedia (India) and Microbial type culture collection and Gene Bank, IMTECH, Chandigarh, India, respectively. The bacterial cultures were maintained in nutrient agar at 4 °C and as 50% glycerol stocks at − 80 °C for long-term storage.

#### Agar well diffusion assay

The antimicrobial activity of the *Sivanar Amirtham* was determined using the agar well diffusion method, followed by enumeration of colony-forming units. One to two colonies of the reference strains from the freshly grown culture plates were inoculated into peptone water and were incubated for 4 h at 37 °C. After the incubation period, the turbidity in the peptone culture was adjusted to 0.5 McFarland standard to achieve about 1.5 × 10^8^ CFU/mL. Sterile swab sticks were inserted into each of the McFarland standard adjusted inoculums, and the excess suspension was squeezed at the walls of the test tubes. The swab stick was spread thoroughly on Mueller–Hinton (MH) agar by lawn culture method and wells were bored in the MH agar plates using a sterilized borer. A stock solution of *Sivanar Amirtham* at the concentration of 50 mg/mL was prepared freshly before each experiment. Into the wells, 100 μL of the various concentrations (2.5 mg/mL, 5 mg/mL, 10 mg/mL, 15 mg/mL, 20 mg/mL, and 25 mg/mL) of the *Sivanar Amirtham* samples were aliquoted and the MH agar plates were incubated at 37 °C overnight. The ZOI around the wells was measured in millimeters (mm) at the end of the incubation period and the ability of the formulation to inhibit the growth of bacteria was evaluated^35^.

#### Colony forming units (CFU) enumeration

The ability of *Sivanar Amirtham* formulation to inhibit the growth of both gram-positive and gram-negative bacteria at various concentrations (2.5 mg/mL, 5 mg/mL, 10 mg/mL, 15 mg/mL, 20 mg/mL, and 25 mg/mL) was evaluated by enumeration of colony forming units. About 100 μL of MH broth containing the *Sivanar Amirtham* at the concentrations tested were aliquoted to the wells of a 96-well microtitre plate. To this, 10 μL of the respective microorganisms at a concentration of 1 × 10^6^ CFU mL^−1^ were inoculated. The microtitre plates were incubated at 37 °C overnight, statically. After the incubation period, the growth media was serial diluted using a sterile 0.9% saline solution and spread-plated on MH agar. The plates were again incubated at 37 °C overnight. The bacterial colonies grown on the agar plates were counted manually and the colony-forming units (CFU) were enumerated^36^.

### In-vitro anti-tuberculosis activity of Mercuric-sulphide based Metallopharmaceutical *Sivanar Amirtham*

#### Bacterial strains and growth conditions

Standard reference strain *Mycobacterium tuberculosis* ATCC 27294 (vaccine strain-H37 RV strain) was obtained from Maratha Mandal’s Central Research Laboratory, Belgaum, India. Then, the bacteria were rejuvenated for 4 weeks and subsequently suspended and diluted equivalent to Mc Farland 0.5 standard turbidity.

#### Microplate Alamar Blue Assay (MABA)

Microtitre Alamar Blue Assay was performed to determine the potential activity of *Sivanar Amirtham* against the *M. tuberculosis* strains. The bacterial growth was detected using the Alamar blue reagent and the change in colour of the reagent was directly related to the activity of the product. Initially, the *M. tuberculosis* strain was revived on Middle brook 7H9 agar, then the scrapping of freshly grown colonies was transferred into Middle brook 7H9 broth and incubated for 24 h at 37 °C. After incubation, the turbidity of the broth was adjusted to 0.5 McFarland standard. The anti-tuberculosis drugs such as Pyrazinamide, Ciprofloxacin, and Streptomycin were used as a positive control for comparison. The metallodrug formulation test sample was prepared by dissolving *Sivanar Amirtham* in sterile water by ultrasonication. Accurately, 200 µL of sterile water was added to the outer perimeter walls of the 96 well-plate to avoid the evaporation of the medium during incubation. About 100 µL of 7H9 broth was added to each well and 100 µL of test sample solution was added to the first well, and then further serial dilutions were made to attain the desirable concentrations (0.2–100 µg/mL). Then the plates were covered and sealed with parafilm and incubated at 37 °C for five days. After incubation, 25 µL of freshly prepared 1:1 mixture of Alamar Blue reagent and 10% Tween 80 was added to the plate and incubated for 24 h. Once the incubation was completed, the 96-well plate was observed for the colour change. The wells with the blue colour indicated the inhibition of growth, whereas the pink denoted the growth of the microorganisms. The MIC was defined as the lowest concentration of the sample, which prevents the colour change to pink from blue^37,38^.

### In-vitro anti-HIV activity of Mercuric-sulphide-based Metallopharmaceutical *Sivanar Amirtham*

#### Cells and viruses

Human T-lymphocyte cells C8166 were donated by the AIDS Reagent Project, the UK Medical Research Council (MRC), and were initially cultured in RPMI-1640 medium with FBS (10%), Streptomycin (100 μg/mL), and Penicillin G (100 units/mL). Laboratory-adapted strain HIV-1_IIIB_ was contributed by the AIDS Research and Reference Reagent Program, National Institute of Health (NIH), China.

#### Anti-HIV study by syncytia inhibition method

Anti-HIV activity was determined from the repressive efficiency of the *Sivanar Amirtham* sample on syncytia formation of HIV-1-induced cells. Initially, 4 × 10^4^ C8166 cells were infected with HIV-1_IIIB_ and these cells were cultured in 96 well plates at 5% CO_2_ for 3 days at 37 °C. After completion of 3 days, the syncytia formation in each well was numbered using an inverted microscope to evaluate the cytopathic effect. The inhibitory effect of the sample was predicted from the resultant Inhibition ratio calculated from the formula and the IC_50_ value (obtained from the plot of inhibition ratio vs. concentration), which depicted the concentration of the test sample that inhibited 50% of the syncytia formation^39^.

$$Inhibition \, ratio \, \left(\%\right)=1-\frac{Number \,of \,syncytia \, in \, test}{Number \,of \,syncytia \,in \,the \,positive \, control}\times 100$$

### In-vitro Haemolysis assay

Haemolytic activity of the metallopharmaceutical *Sivanar Amirtham* was investigated using the pooled blood obtained from 8 to 12 weeks old Wistar rats (*Rattus norvegicus*), with prior approval from the institutional animal ethical committee (742/SASTRA/IAEC/RPP) the surgical procedures were approved by IAEC at SASTRA University. The experimentations were executed ensuing the guiding principle notified by the Committee for the Purpose of Control and Supervision of Experiments on animals (CPCSEA), Ministry of Environment and Forest, Government of India and Animal Research: Reporting of In Vivo Experiments (ARRIVE). The blood collected in an Ethylene diamine tetra acetic acid (EDTA) tube was centrifuged to separate plasma and erythrocytes. The isolated erythrocytes were washed with saline and separated using centrifugation (Remi R24, Remi Elektrotechnik Ltd, India) for 10 min at 3000 rpm, repeatedly three times. The pellet was used to prepare 1% erythrocyte solution using saline. The *Sivanar Amirtham* sample solution was prepared using saline at different concentrations (100, 150, 200, 250, and 300 µg/mL), based on the recommended human dose (100 mg). For each 1 mL of *Sivanar Amirtham* sample solution, 1 mL of the erythrocyte solution was added and the solution mixtures were mixed using a rotary laboratory shaker (Stuart Equipment, USA) at room temperature for one hour at 100 rpm, followed by centrifugation at 3000 rpm for 15 min. The obtained supernatant was collected to quantify the amount of haemoglobin released using a spectrophotometer at 540 nm. Triton X-100 solution (0.1%) and normal saline were considered as the positive and negative controls, respectively^40^. The experiment was carried out in triplicate and the haemolytic index was calculated using the formula,

$$\mathrm{Haemolytic \, Index}=\frac{\mathrm{AbS}-\mathrm{AbN}}{\mathrm{AbP}-\mathrm{AbN}}$$

where *AbS* absorbance of sample tested, *AbN* absorbance of negative control, *AbP* absorbance of positive control.

### Statistical analysis

The experimental data obtained from different assays were subjected to statistical analysis. The results of CFU enumeration were analysed by one-way ANOVA followed by Dunnett’s multiple comparison tests with significance set at *p* < 0.05, using GraphPad Prism version 8.0.2 (GraphPad Software Inc., CA, United States)^41^. The other results such as the zone of inhibition (mm) by agar well diffusion assay, syncytia inhibition assay, and haemolytic assay were limited to mean (n = 3) with standard deviation (error bars)^42^.

## Results

### Analytical characterization studies of Mercuric-sulphide-based Metallopharmaceutical *Sivanar Amirtham*

#### Size and surface morphology

**Figure 1:**
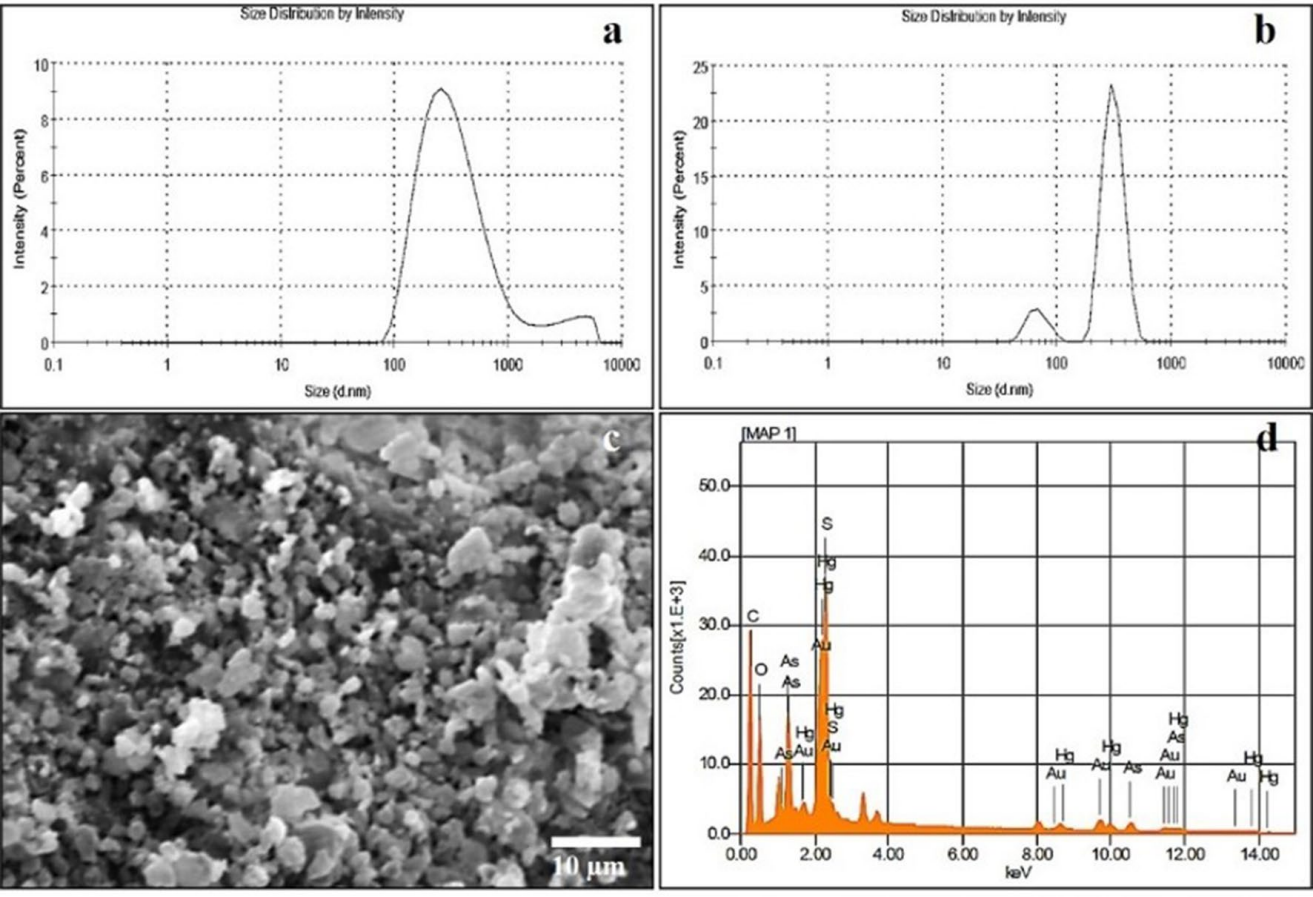
Particle size distribution of (**a**) *Kajjali*, (**b**) *Sivanar Amirtham* and (**c**) SEM and (**d**) EDAX image of the metallopharmaceutical formulation.

*Sivanar Amirtham* viewed under FE-SEM at 1000× magnification displayed the images with irregularly shaped micron and nano-sized particles (Fig. 1). The particles were clustered and agglomerated because of the influence of mechano-chemical grinding during the preparation. The EDAX analysis confirmed the uniform distribution of mercury, sulphur, and arsenic in the formulation. The average particle size of *Kajjali* (mercuric sulphide), the intermediate product formed during the metallopharmaceutical preparation was found to be 284 nm with a polydispersity index (PDI) of 0.301, which confirmed the uniform mono disperse nano-sized metal–mineral complex particles. However, the final product of *Sivanar Amirtham* displayed an average particle size of 758 nm with a PDI value of 0.749, indicating the mixture of both micron and nano-sized particles, which could be attributed to the addition of more fibrous seeds and rhizome powder ingredients during the formulation processing^34^. The poly-disperse characteristics of the final formulation depicted the existence of more than one type of material, due to the addition of metals and minerals in combination with different herbal powders of seeds and rhizomes^43^.

#### FTIR analysis

**Figure 2:**
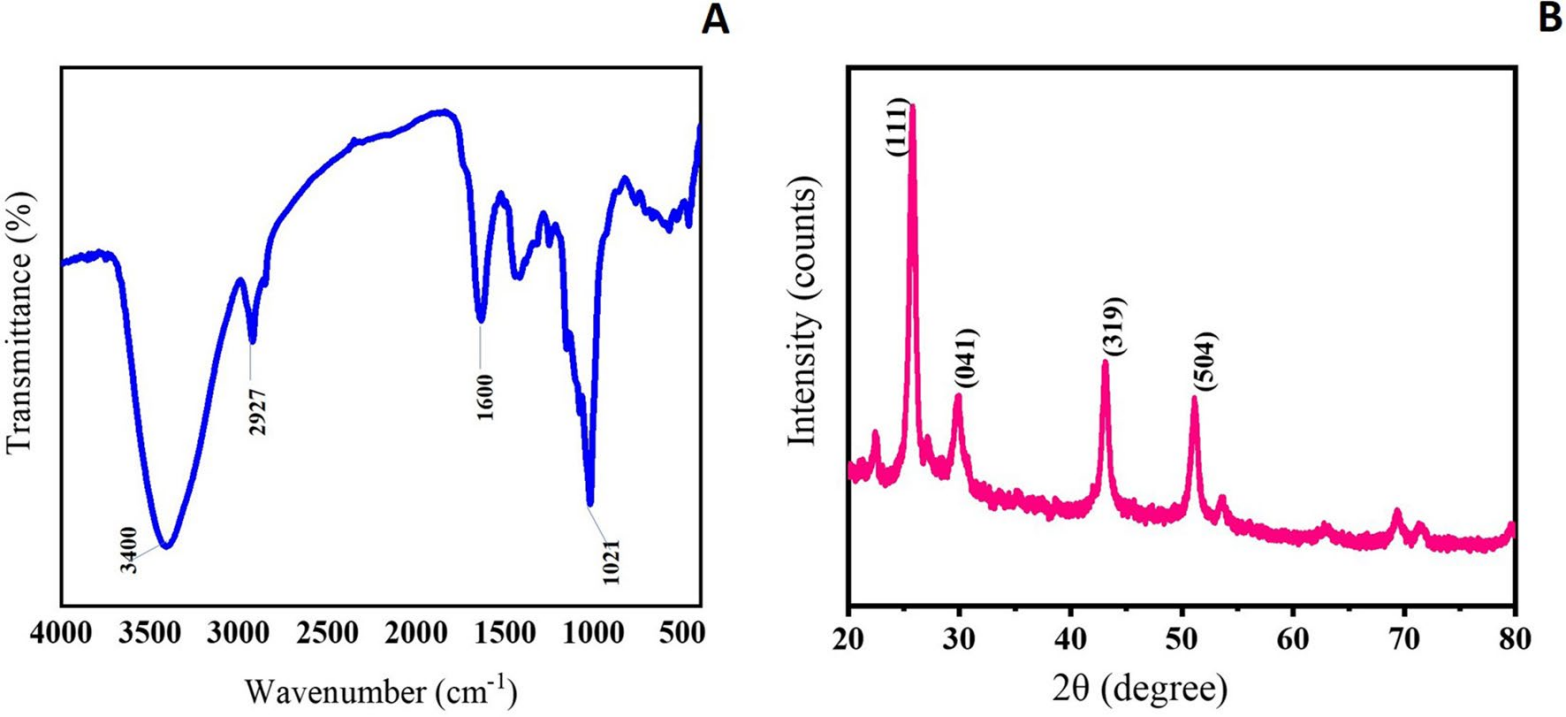
(**A**) FTIR spectrum and (**B**) XRD pattern for mercuric-sulphide-based metallopharmaceutical *Sivanar Amirtham.*

The FTIR spectrum of *Sivanar Amirtham* explored the characteristic functional groups and identified the chemical nature of the product (Fig. 2). The prominent peaks identified at around 1021 cm^−1^ represented the presence of mercuric sulphide. The peak at 763 cm^−1^ signified the presence of the B–O–B bending vibration of borax. The S–H stretching vibration was observed at 2927 cm^−1^ and the peak at 576 cm^−1^ in the spectrum was related to arsenic trisulphide. The peaks observed at 3410 cm^−1^, 1634 cm^−1^, 1417 cm^−1^, 1152 cm^−1^, and 1078 cm^−1^ indicated –O–H stretching, –C=N stretching, –C=C aromatic stretching, –C–O stretching of ether and alcohol groups, respectively, which could be due to the presence of the organic phyto compounds. The functional groups identified in the FTIR spectrum convincingly represented the presence of a metal–mineral complex along with the herbal ingredients in the *Sivanar Amirtham* formulation^34,44–46^.

#### XRD analysis

The XRD pattern of the *Sivanar Amirtham* displayed the semi-crystalline nature of the formulation, which could be attributed to the changes in crystal behavior and physical forms of metals and minerals by the addition of herbal ingredients in the product (Fig. 2). The characteristic intense peaks were observed at the angles of 25.78°, 43.09°, 51.14°, 29.86° and 22.45, indicating the presence of metals and minerals. The sharp peaks revealed from the XRD spectrum at 2θ values of 26°, 43°, and 51° represented the crystalline form of Mercuric sulphide (β-HgS/metacinnabar) present in cubic phase (JCPDS No. 06-0261). The existence of other minor peaks at 15° and 23° denoted the presence of red orpiment and arsenic trisulphide (JCPDS No. 24-0078) and sulphur (JCPDS No. 88-2600), respectively^34,47^.

#### XRF analysis

The elemental composition of the *Sivanar Amirtham* was determined using XRF analysis since the presence of mercury, sulphur, red orpiment (arsenic trisulphide), and their oxides form influence the stability and activity of the product^34^. The XRF results revealed the elemental composition of mercury (Hg), sulphur (S), and arsenic trisulphide (As_2_S_3_) as 0.87%, 78.84%, and 0.58% respectively, which were the primary metal and mineral ingredients. The absence of the oxide form of mercury was evident in Table 1. The percentage of oxide forms of sulphur and arsenic were 70.19% and 0.76%, respectively. Even though the presence of arsenic trioxide was below 1%, it could exhibit specific medicinal properties, which have been approved by the FDA as a therapeutic agent for anti-cancer treatment^48,49^. The results evidenced the presence of mercuric sulphide and also ensured the negligible amount of elemental form of Hg (< 0.87%). Moreover, it was proved that Hg was not converted to HgO, which could elicit a high risk of toxicity due to its highly bio-accessible nature. Arsenic was present in the form of arsenic trisulphide, which was insoluble in water and dilute HCl, and this ensured reduced toxicity due to its less absorbable form^50^. All the other minerals and their oxides identified could be due to the traces from the herbomineral composition, nevertheless, they are considered to be present in insignificant amounts.

**Table 1:** XRF analysis for the elemental composition of mercuric-sulphide based metallopharmaceuticals *Sivanar Amirtham.*

Elements in oxide form		Element form	
Formula	Concentration (%)	Formula	Concentration (%)
SO_3_	70.19	S	78.84
Hg	0.87	Hg	0.87
As_2_O_3_	0.76	As	0.58
K_2_O	5.11	K	4.24
Al_2_O_3_	0.65	Al	0.34
Na_2_O	8.20	Na	6.08
CaO	3.93	Ca	2.81
MgO	4.36	Mg	2.63
SiO_2_	2.56	Si	1.20
Cl	1.51	Cl	1.51
P_2_O_5_	1.55	P	0.68
TiO_2_	0.08	Ti	0.05
Fe_2_O_3_	0.08	Fe	0.06
MnO	0.07	Mn	0.05

### In-vitro anti-microbial activity of Mercuric-sulphide based Metallopharmaceutical *Sivanar Amirtham*

#### Agar well diffusion assay

**Figure 3:**
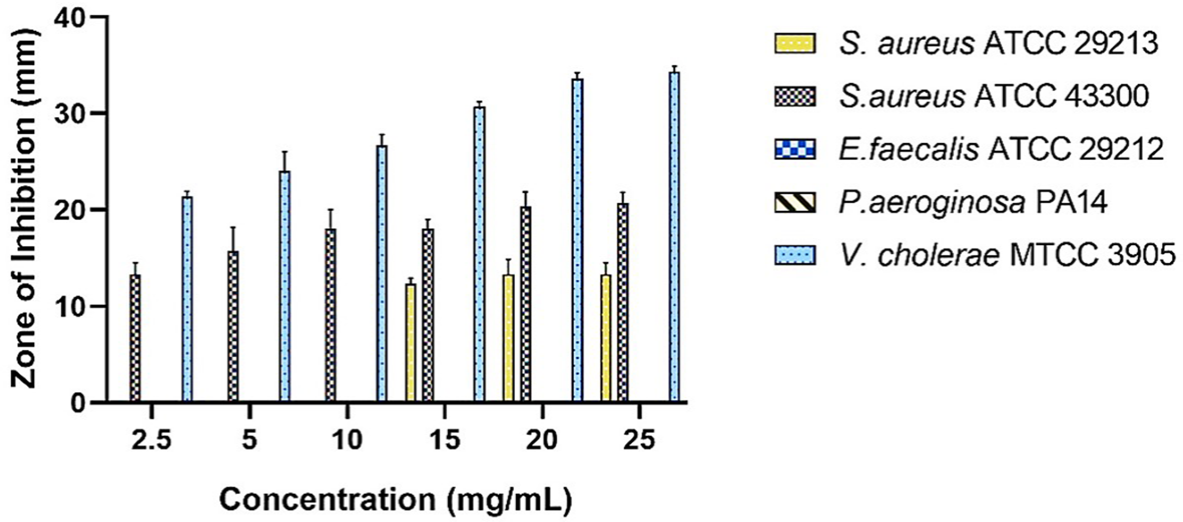
Antimicrobial activity of Mercuric-sulphide based metallopharmaceutical *Sivanar Amirtham* against Gram-positive and Gram-negative bacterial pathogens.

The agar well diffusion assay results indicated that *Sivanar Amirtham* was effective in inhibiting the growth of *S. aureus* and *V. cholerae*. When tested against Gram-positive *S. aureus* MSSA, the formulation inhibited the growth effectively and the zone of inhibition (ZOI) was observed to be 12.3 ± 0.6 mm, 13.3 ± 1.5 mm, and 13.3 ± 1.2 mm at the higher concentrations of 15 mg/mL, 20 mg/mL and 25 mg/mL, respectively. However, in the case of the MRSA reference strain, *Sivanar Amirtham* was more effective in inhibiting the growth of Gram-positive cocci even at lower concentrations (2.5 mg/mL, 5 mg/mL, 10 mg/mL). The growth inhibitory activity of the drug was evident as an increased ZOI was observed at higher concentrations. However, the drug exhibited the most significant antimicrobial activity against the Gram-negative bacterial pathogen *V. cholerae*. When tested against *V. cholerae*, the ZOI increased dose-dependently from lower to higher concentrations from 21 to 34 mm. Interestingly, the formulation did not show any antibacterial activity against other bacterial pathogens including *Enterococcus faecalis* and *Pseudomonas aeruginosa* tested (Fig. 3; Supplementary Fig. 1, Supplementary Table 1).

#### Colony forming units

**Figure 4:**
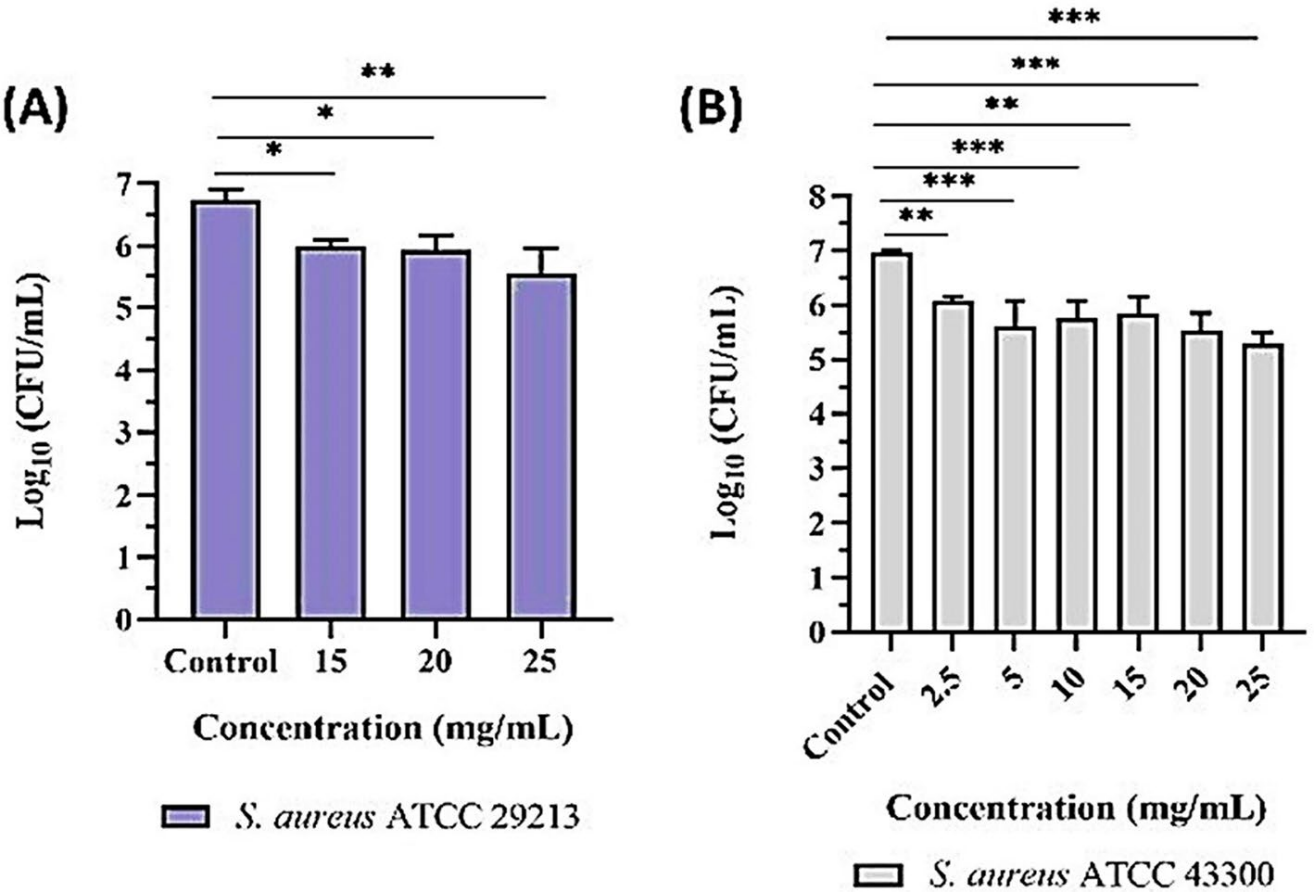
Colony forming unit enumeration assay for (**A**) *S. aureus* MSSA strain and (**B**) MRSA strains treated with mercuric-sulphide based metallopharmaceutical formulation (significant difference is indicated by *p < 0.05; **p < 0.01; and ***p < 0.001).

The agar well diffusion assay revealed that both strains of *S. aureus* were susceptible to *Sivanar Amirtham* at various concentrations. Enumeration of the colony-forming units (CFU) of MSSA and MRSA at the effective concentrations showed that the sample had reduced the CFU of both *S. aureus* strains. When tested against MSSA, there was a slight reduction in CFU at higher concentrations when compared to untreated *S. aureus* (Fig. 4A). Comparison between treated and untreated MRSA showed a significant reduction (p < 0.05) in the CFU of bacteria at the concentrations tested (Fig. 4B). Similarly, the enumeration of CFU of *V. cholerae* revealed that there was complete inhibition of CFU on all the concentrations tested when compared to the untreated *V. cholerae*. This formulation was considered to be a safer medicine, based on its complexation between metal, mineral, and herbal conjugates. Moreover, the in-vitro and in-vivo toxicity studies had shown that *Sivanar Amirtham* had exhibited only selective toxicity toward cancerous cells^43^. However, limited resources are available reporting the beneficial anti-microbial properties of *Sivanar Amirtham*. The present study results explored and evidenced the significant potential anti-microbial properties of the traditional Siddha medicine preparation against MRSA and *V. cholera.*

### In-vitro anti-tuberculosis activity of Mercuric-sulphide based Metallopharmaceutical *Sivanar Amirtham*

#### Microtitre Alamar blue assay

**Figure 5:**
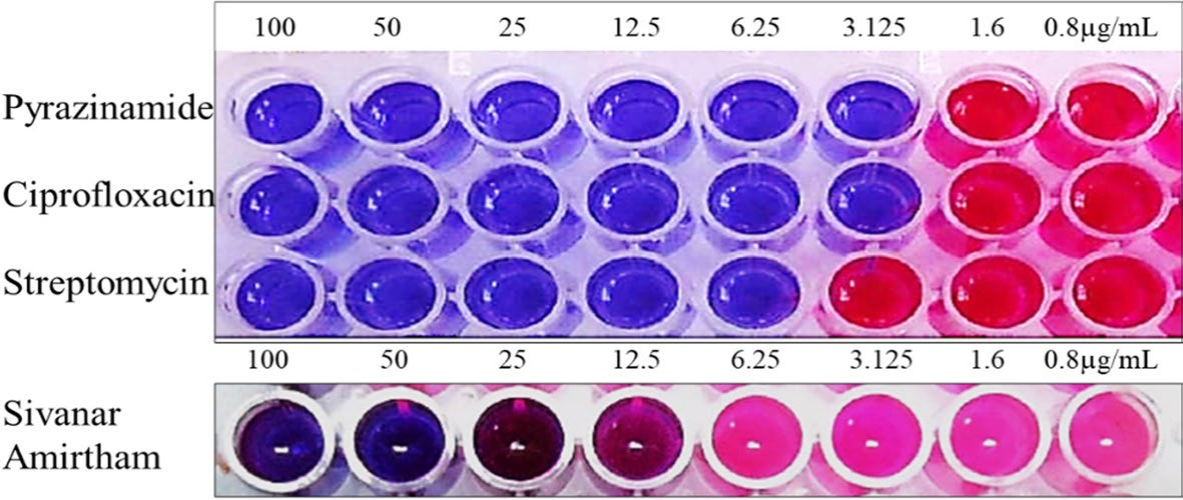
Anti-TB activity of mercuric-sulphide based metallopharmaceutical *Sivanar Amirtham* against *M. tuberculosis* by Alamar Blue assay.

The anti-mycobacterial activity of *Sivanar Amirtham* was assessed by perceiving the metabolic activity of the bacteria as an indicator. The change in colour from blue to pink of the Alamar blue indicator showed the growth of *M. tuberculosis* and its resistance (R) towards the test sample, whereas, the retention of blue colour confirmed the susceptibility/sensitivity (S) of the microorganisms. *M. tuberculosis* strains were found to be susceptible to *Sivanar Amirtham* at concentrations of 12.5 µg/mL, 25 µg/mL, 50 µg/mL, and 100 µg/mL. It was found to be comparable with the activity of standard drugs such as Pyrazinamide, Ciprofloxacin and Streptomycin (Table 2; Fig. 5).

**Table 2:** Anti-TB activity of metallopharmaceutical *Sivanar Amirtham* compared to standard drugs against *M. tuberculosis.*

Sample	100 µg/mL	50 µg/mL	25 µg/mL	12.5 µg/mL	6.25 µg/mL	3.12 µg/mL	1.6 µg/mL	0.8 µg/mL
Pyrazinamide	+	+	+	+	+	+	–	–
Ciprofloxacin	+	+	+	+	+	+	–	–
Streptomycin	+	+	+	+	+	–	–	–
*Sivanar Amirtham*	+	+	+	+	–	–	–	–

+ denotes Sensitive, ‘−’ denotes resistant.

### Anti-HIV activity of Mercuric-sulphide based Metallopharmaceutical *Sivanar Amirtham*

**Figure 6:**
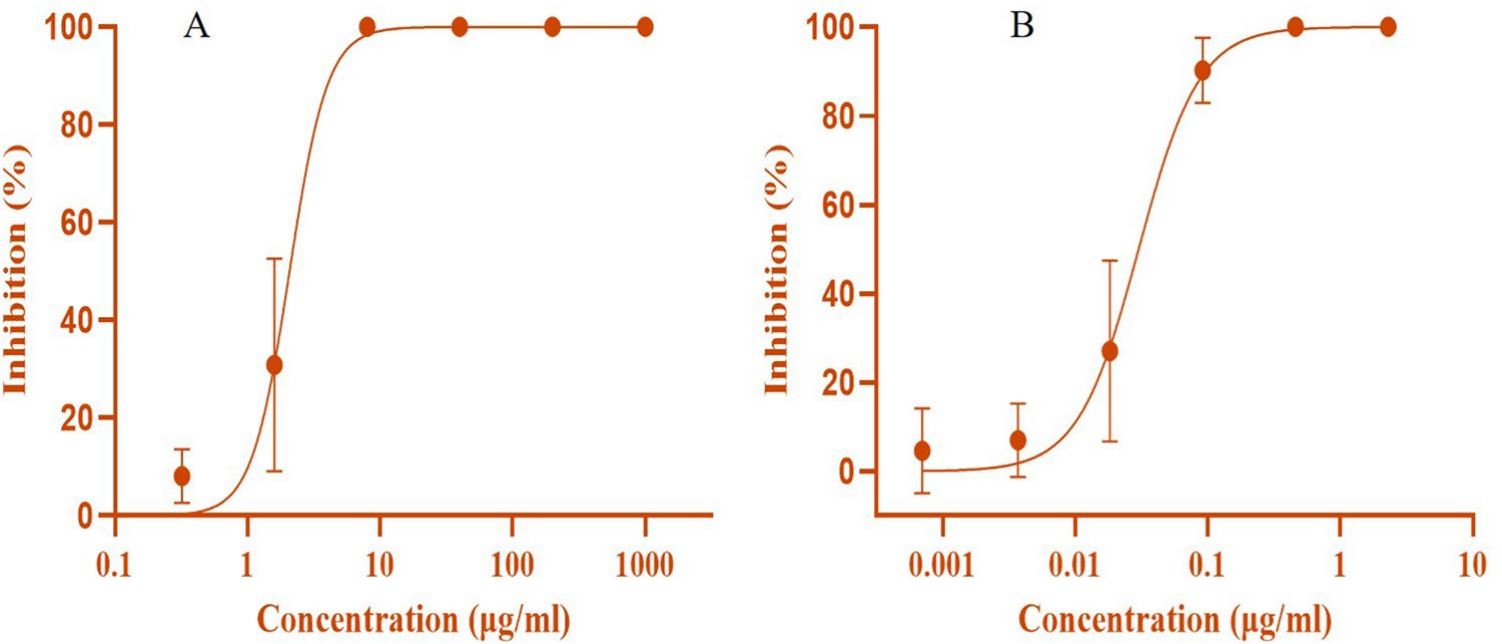
Anti-HIV activity of mercuric-sulphide-based (**A**) metallopharmaceutical *Sivanar Amirtham*. (**B**) Standard drug Lamivudine assessed by Syncytia inhibition assay.

From the syncytium inhibition assay, the IC_50_ value of the standard anti-HIV drug Lamivudine and the mercuric-based nano-herbomineral formulation was found to be < 0.1 μg/mL and ≈ 5 μg/mL, respectively (Fig. 6). The results provide evidence for the anti-HIV activity of the mercuric-sulphide-based nano herbomineral formulation *Sivanar Amirtham* against C8166 cell lines infected with HIV-1_IIIB_ viral strain, as compared to the standard antiretroviral drug. SA could be considered as a potential alternative and adjuvant that can be used for prophylactic and therapeutic applications for antiretroviral therapy since the obtained IC_50_ value was significantly better than the previously published reports of other metal-based pharmaceuticals^51,52^.

### In-vitro Haemolysis assay

**Figure 7:**
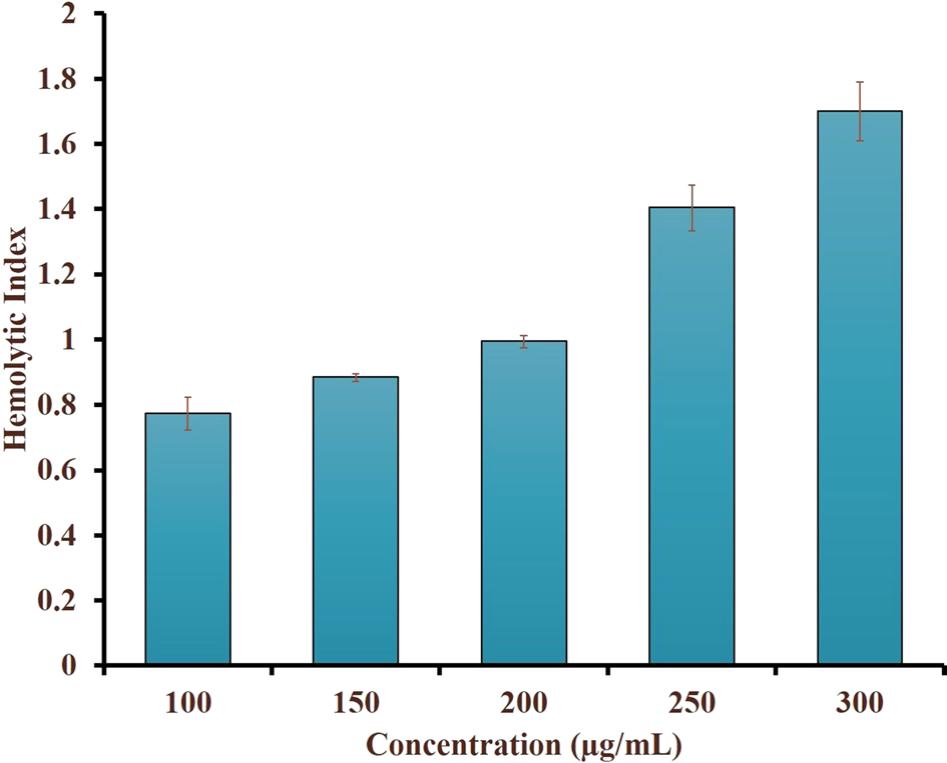
Haemolytic effect of the metallopharmaceutical *Sivanar Amirtham.*

The in-vitro haemolytic activity was assessed to identify the toxicity profile of *Sivanar Amirtham*, using the erythrocytes of Wistar rats. The haemolysis index at different concentrations of the samples viz., 100, 150, 200, 250, and 300 µg/mL was found to be 0.77, 0.88, 0.99, 1.4, and 1.7, respectively. The haemolytic effect induced by the metallopharmaceutical formulation was found to be concentration-dependent (Fig. 7). However, the highest haemolysis index outcome was < 2 at the concentration of 300 µg/mL, which proved that *Sivanar Amirtham* could be considered as a non-haemolytic product.

## Discussion

The metal-complexed drugs have captivated a lot of interest due to their high therapeutic potential to manage the antibiotic resistance threats faced worldwide. As the use of metal-based pharmaceuticals has been considered as an alternative strategy for the antibiotic resistance crisis, the already existing Siddha medicine, *Sivanar Amirtham* containing metal-mineral complex has been assessed in the present research to validate for scientific proof-of-evidence. The size of the particles had an influential role in the activity of the formulation, as they were directly encountered with the cell surface of the microbes. This attribution was evidenced by increasing antimicrobial activity with a decrease in the size of the metallic entities. The smaller particles easily and effectively penetrate and interact with the microbial membrane. The metal–mineral complex, *Kajjali* in *Sivanar Amirtham* had nano-sized particles, which played a key role in microbial toxicity^53–55^. Also, the antibacterial action of organic entities in the herbals was enhanced by the detoxified minerals and other inorganic residues present in the metallopharmaceuticals^56^. Recently, *Abhrak bhasma*, an ayurvedic metal–mineral complex showed particle size distribution in the range of 50–500 nm^57^, which had a major impact on the antibacterial activity of the formulation^58^. Similarly, other traditional preparations like *Mandura bhasma* and *Vanga bhasma* possessed average particle sizes between 200–300 nm and 150–300 nm, respectively^59^. The *Mandura bhasma* had shown strong antibacterial activity against *S. aureus*, *P. aeruginosa*, *K. pneumoniae*, *S. typhi*, *E. coli*, *E. aerogenes*, *S. epidermidis,* and *S. typhimurium*^60^. Likewise, *Vanga bhasma* was proved to be effective against *S. aureus*, *C. albicans*, *B. subtilis*, *E. coli,* and *K. pneumoniae*^61^.

*Staphylococcus aureus* is responsible for multiple infections from wounds to post-surgical infections, and the MRSA strains show resistivity against antibiotics including penicillin, tetracycline, methicillin, erythromycin, and vancomycin^62–64^. *Sivanar Amirtham* had shown activity against *S. aureus* (ATCC 29213) at higher concentrations of 15 mg/mL, 20 mg/mL, and 25 mg/mL with a ZOI of 12.3 ± 0.6 mm, 13.3 ± 1.5 mm, and 13.3 ± 1.2 mm, respectively. As per the CLSI standards, the resultant ZOI categorized the medicine under the sensitivity (≥ 12) region, when compared with the standard drug Vancomycin (30 μg/disk), commonly used for treating *S. aureus* infections^65^. *Sivanar Amirtham* had shown strong antibacterial activity in a dose-dependent manner from 2.5 to 25 mg/mL towards Methicillin-Resistant *S. aureus* (ATCC 43300), as the ZOI increased from 13.3 ± 1.2 to 20.7 ± 1.2 mm, correspondingly. According to the results, *Sivanar Amirtham* possessed highly significant antibacterial activity against MRSA than *S. aureus*. Since the MRSA strains are the major concern regarding microbial resistance, these results provided scientific evidence of the potential antibacterial activity, therefore *Sivanar Amirtham* could be considered as a potential alternative to the single antibiotic treatment. In the similar fashion, Wijenayake et al*.* had reported the antimicrobial activity of herbomineral formulations *Arogyawardhana Vati* and *Manikya Rasa* against *P. aeruginosa, E. coli, S. aureus*, MSRA, and *C. albicans*. These products contain mineral and metal constituents such as cinnabar, iron, biotite, sodium, potassium, calcium, magnesium, zinc, copper, and arsenic. The potential activity of the medicine against gram-positive and gram-negative bacteria was evident based on the contribution of organic and inorganic constituents in the formulations. Likewise, *Sivanar Amirtham* comprised of mercury, sulphur, red orpiment, and borax as the major metal and mineral ingredients along with polyherbals such as *Z. officinale*,* P. longum*,* P. nigrum*,* D. filixmas*, and *A. ferox*. So, it could be inferred that the metal–mineral complex along with the organic content of herbals in the metallopharmaceuticals synergistically enhanced the antibacterial property. Metal ions are predicted to form coordination bonds with nitrogen, oxygen, and sulphur atoms that are abundant in biomolecules and organic compounds, which could exhibit broad-spectrum antimicrobial activity^66^. Previously reported studies had suggested that the release of metal ions from the formulation could play a key role in microbial toxicity, and the therapeutic efficacy could be directly related to the detoxified metal–mineral complex^56^. *Padikara Parpam* had been reported with a ZOI of 14 mm at 4 mg/mL concentration^67^ and *Mupoora Chendooram* was identified with a ZOI of 10 mm at the concentration of 25 mg/mL^22^ against *S. aureus.* Similarly, *Mrityunjay Rasa* sample showed a ZOI of 20 mm^68^, *Thazhampoo Mathirai* displayed a ZOI of 16 mm^69^, *Kanagalinga Mezhugu* presented a ZOI of 15–19 mm^70^. However, all these results showing ZOI against *S. aureus* strains were comparatively lesser than the potential activity of *Sivanar Amirtham*. *Mahavat Vidhwansan Rasa* had been reported with ZOI as 21 mm at a concentration of 100 mg/mL against MRSA strains^71^, however, it was significantly lesser than the prepared *Sivanar Amirtham* formulation showing ZOI of 20.7 mm at 25 mg/mL concentration. Sham prasad et al*.* reported a similar case of metallic nanoparticles functionalized with secondary metabolites, which were studied for their activity against MRSA. Kaempferitrin (a secondary metabolite extracted from *Crotalaria juncea*) conjugated with elemental silver and copper showed a significant reduction in microbial bioburden with enhanced antimicrobial and anti-biofilm activity against Methicillin-resistant *Staphylococcus aureus*. Synthesizing metallic nanoparticles capped with phytochemicals had a prominent role to combat drug resistance against several microbial strains. It was reported that the metal ions could stick to the bacterial cell wall using electrostatic attraction and penetrate the cells resulting in cell death^72^. These results provided supportive information to explain the possible mechanisms of action to *Sivanar Amirtham*.

*Vibrio cholerae* causing the acute infectious disease cholera, remains a serious threat for the maximum percentage of mortality in developing countries. *V. cholerae* could change between planktonic and biofilm lifestyles^73^. So, developing a bioconjugate is considered necessary to fight against resistant strains. *Sivanar Amirtham* showed the most significant activity against *V. cholerae* with the highest ZOI at 34.3 mm and the lowest at 21.3 mm. While comparing with the zone diameter of Azithromycin (≥ 18 for 15 μg/disk) and Ciprofloxacin (≥ 21 for 15 μg/disk) as per CLSI standards^65^, *Sivanar Amirtham* exhibited significant activity with higher ZOI. This confirmed that the formulation was sensitive like other antibiotics used to treat cholerae and would be a promising drug for efficient treatment against resistant strains also. Interestingly, the susceptibility of *V. cholerae* against Ciprofloxacin (ZOI 30–34 mm) reported by Christan et al*.*, was found to be equivalent to *Sivanar Amirtham* (ZOI 30.6–34 mm). Some other herbomineral formulations showing similar activity against *V. cholerae* were *Mupoora Chendooram* (ZOI 17 mm)^22^ and *Kanagalinga Mezhugu* (ZOI 14–19 mm)^70^. Salem et al*.* reported the effective antibacterial activity of silver and zinc nanoparticles against *V. cholerae*^74^. However, *Sivanar Amirtham* did not show any ZOI for *E. faecalis and P. aeruginosa*. The activity of the metallo drugs toward the microorganisms could be linked to the variations in the morphological characteristics between gram-positive and gram-negative microorganisms^75^. Herbomineral formulations like *Mrityunjay Rasa* and *Mahavat Vidhwansan Rasa* also showed no activity against *P. aeruginosa*^68,71^. This might be due to the surface charge and the electrostatic attraction between the metal complexes with microbes. Drugs with positively charged particles could readily adhere to the negatively charged bacterial cell wall. However, mercuric sulphide was found to be negatively charged particles, which perhaps be the factor contributing to its inactivity over gram-negative bacteria^76,77^. On the contrary, its inactivity against gram-positive bacteria could be due to its dissimilarity of permeable peptidoglycan layer, which is another deciding factor for the entry of drug into the microorganisms^58^. Also, other than penetration, various modes of action influence the potential of nanoparticles to exert antibacterial activity. This includes interaction with intracellular contents including DNA and proteins, generation of ROS, and disruption of bacterial cell walls by the nanoparticles to exert the site-specific therapeutic efficacy. The specific activity of the herbo-mineral formulation against the pathogens requires a detailed study of the mechanism of action, which is the scope of our future study.

In-vitro anti-mycobacterial activity was attempted for *Sivanar Amirtham* through Alamar blue assay to study the minimum inhibitory concentration required against *M. tuberculosis*^78^. The MABA results showed that the metallopharmaceutical was sensitive at concentrations from 12.5 to 100 µg/mL. Several herbal medicines possessed comparable anti-tuberculosis activity along with immune stimulant properties viz., the extracts of *Allium sativum* (garlic) and *Zingiber officinale* (ginger) screened against *M. tuberculosis* H37RV strains showed a 2% minimum killing rate and 4% against multi-drug resistant strains of the same, which could alleviate the symptoms and manifestations of tuberculosis; additionally, *Spondias cytherea* (kedodong) extract in combination with Rifampicin had shown increased activity^79^. Therefore, combinational therapy of herbals and herbominerals along with antibiotics could be repurposed for treating several infectious diseases in a more effective way.

The presence of secondary metabolites including alkaloids, flavonoids, phenolics, saponins, steroids, and triterpenoids that existed in the herbal ingredients like *Z. officinale*,* P. nigrum*,* P. longum*,* D. filixmas* and *A. ferox* could be another valuable reason for the potential antibacterial effect. The phenolic compounds kill microorganisms by protein denaturation, as the compounds form hydrogen bonds with proteins causing damage to the protein structure in a bacterial cell that distresses the cytoplasmic membranes and permeability of the cell wall. This results in cell lysis due to the imbalance in ionic and macromolecular components. Saponins affect the surface tension of bacterial cell walls and lead to lysis by damaging the cell membrane permeability. Interference of alkaloids with peptidoglycan constituents causes cell death due to incomplete formation of the cell walls. As steroids are lipophilic compounds, they can permeate the phospholipid cell membrane, whose interaction leads to the brittle cell membrane and finally lysis of cells^79^.

Although the mechanism of action of metallopharmaceuticals over the microbes was not explored, the possibility of exhibited activity through multi-level therapeutic effects is demonstrated based on the chemical composition. The expected ways of these metallodrugs to affect the pathogens were by disrupting the cell wall, causing oxidative reactions by producing ROS, and deactivation proteins^80^. Therefore, metal-based polyherbal drugs are considered more efficient through the multiple targeted actions, which makes it difficult for the pathogens to develop resistance, unlike other chemical antibiotics.

The syncytium inhibition assay of *Sivanar Amirtham* had shown 50% inhibitory action against syncytia formation of HIV-1_IIIB_ at ≈ 5 µg/mL. Formerly research findings on metal/mineral-based pharmaceuticals had shown anti-HIV activity at very high concentrations. For instance, Song et al*.* reported similar results for metalloporphyrin, wherein manganese complex and iron complex had shown IC_50_ > 200 µg/mL against MT-4 cell lines and HIV-1_IIIB_^51^. Some sulfated polysaccharides like dextrin sulfate and λ-carrageenan exhibited the inhibitory effect at > 500 µg/mL and > 625 µg/mL, respectively^52^. The zinc group of metals like zinc acetate and zinc chloride inhibited the syncytia formation at 8 µg/mL individually, while zinc nitrate exhibited IC_50_ at 13 µg/mL^27^. Although cadmium acetate (0.18 µg/mL) and mercury chloride (0.12 µg/mL) showed very mild activity at lower concentrations, they hardly affected the HIV-1 antigen in C8166 cells. However, a strong inhibition of syncytia formation was observed in this high concentration^27,30^. In our experiments, *Sivanar Amirtham* had shown notable anti-HIV activity at very low concentrations due to the presence of mercury in a complex form with sulphur, along with other minerals and herbs. Savarino et al*.* provided the evidence with patented Auranofin, wherein gold metal complex reactivated the latent HIV-1 and counteracted with the cellular machinery (like thioredoxin, thioredoxin, and glutathione) by inducing selective killing of infected cells with limiting effect over the normal cells. These compounds were also referred as epigenetic modulators/epigenetic metallodrugs, which had the ability to target only the infected cells^81^. In this context, it could be evidenced that *Sivanar Amirtham* could be used as an adjuvant along with other anti-HIV drugs, to produce a synergistic effect to kill the latent HIV cells and also act as an epigenetic modulator, as it contains metal-complexes in biosimilar form with poly herbal ingredients that possessed significant anti-oxidant activity. Though previous studies have reported the anti-HIV activity of certain metals and metal complexes through the syncytium inhibition assay, our study has attempted to estimate the syncytium inhibition activity of the traditional metallopharmaceutical, *Sivanar Amirtham*. Further, molecular-level studies could be performed for a thorough understanding of the mechanism of HIV inhibition.

The in-vitro haemolysis assay has been considered as a direct indication of toxicity of the drug formulations intended for use in contact with blood. The similarities of erythrocyte membranes comparable to cell membrane, render them an appropriate choice for studying drug interactions in-vivo. The haemolysis of lipid bilayer membrane of red blood cells could be related to the concentration and the potency of a drug. The ASTM F756-13 standards for the assessment of haemolytic properties of materials had categorized the degree of haemolysis and denoted the grades with haemolytic index values of 0–2 as non-haemolytic, 2–5 as slightly haemolytic and > 5 as haemolytic^82^. Since *Sivanar Amirtham* had shown haemolytic activity in the range between 0.77 and 1.7 for the increasing function of concentration, it falls under the grade (0–2) of non-haemolytic material, which confirmed it as non-toxic and haemo-compatible. The results were found to be comparable to previously reported zinc oxide nanoparticles functionalized with hydroxyapatite and europium, which exhibited haemolysis effect as 1.85^40^.

## Conclusion

The developed metallopharmaceutical formulation *Sivanar Amirtham* was analyzed for its morphological characteristics, presence of active functional groups, state of crystallinity, and elemental composition. *Sivanar Amirtham* had demonstrated prominent antibacterial activity against MRSA and *V. cholera*, and moderate activity against *S. aureus.* In addition, specific anti-tuberculosis activity was evidenced against ATCC 27294 in the broad range of concentration from 12.5 to 100 µg/mL. Further, the metallopharmaceutical repurposing attempt towards anti-viral potential against HIV-1_IIIB_ showed an anti-HIV effect. The comprehensive study of the traditional metallopharmaceuticals for anti-retroviral therapy would help in treating patients with co-epidemic conditions of HIV with TB. The outcome of the results highlighted the scientific evidence for the application of metallopharmaceutical as an effective antimicrobial agent with a non-haemolytic property, which could be proposed as an alternative to antibiotics to combat antimicrobial resistance and multi-drug resistance challenges, as well as a prophylactic and therapeutic adjuvant for anti-viral, especially anti-HIV therapy. The wide range of activity was achieved with the help of a metal–mineral complex by targeting the multiple biomolecules and sites in the bacterial and viral infected cells. Further research could be extended to understand the specific mechanism of action, molecular-level interactions, toxic reactions, and transcriptional and proteomic changes against the pathogens. Consistently, assessing the polyherbomineral formulations for repurposing against antimicrobial and antiviral infections will set a new direction for future therapeutics as well as counteract drug resistance.

### Supplementary Information

Supplementary Information.

## Data Availability

The datasets generated and/or analyzed during the current study are not publicly available due to a funded project and No permission from the same) but are available from the corresponding author at Pharmaceutical Technology Laboratory, ASK-II, Lab No: 214, School of Chemical & Biotechnology, SASTRA Deemed-to-be-University, Thanjavur-613401, Tamil Nadu, India or through email-ramya@scbt.sastra.edu.

## References

[1] Kon K, Rai M (2016). Antibiotic Resistance: Mechanisms and New Antimicrobial Approaches.

[2] Shibl AM, Memish Z, Osoba A (2016). Antibiotic resistance in developing countries antibiotic resistance in developing countries. J Infect. Dis..

[3] Ventola CL (2015). The antibiotic resistance crisis Part 1: Causes and threats. Pharm. Therap..

[4] de Kraker MEA, Stewardson AJ, Harbarth S (2016). Will 10 million people die a year due to antimicrobial resistance by 2050?. PLoS Med..

[5] Koya SF (2022). Consumption of systemic antibiotics in India in 2019. Lancet Reg. Health Southeast Asia.

[6] Indian Council of Medical Research. *Antimicrobial Resistance Research and Surveillance Network *(2021).

[7] Ranjalkar J, Chandy SJ (2019). India ’ s National Action Plan for antimicrobial resistance—an overview of the context, status, and way ahead. J. Fam. Med. Primary Care.

[8] Kumar SG, Roy G (2013). Antimicrobial resistance in India: A review. J. Nat. Sci. Biol. Med..

[9] Mandal J, Dinoop KP, Parija SC (2012). Increasing antimicrobial resistance of Vibrio cholerae O1 biotype El Tor strains isolated in a tertiary-care centre in India. J. Health Popul. Nutr..

[10] Das B, Verma J, Kumar P, Ghosh A, Ramamurthy T (2020). Antibiotic resistance in Vibrio cholerae: Understanding the ecology of resistance genes and mechanisms. Vaccine.

[11] Taneja N, Sharma M (2012). Antimicrobial resistance in the environment: The Indian scenario. Indian J. Med. Res..

[12] Aiyegoro, O. A. & Okoh, A. I. Use of bioactive plant products in combination with standard antibiotics: Implications in antimicrobial chemotherapy (2010).

[13] Anand U, Jacobo-Herrera N, Altemimi A, Lakhssassi N (2019). A comprehensive review on medicinal plants as antimicrobial therapeutics: Potential avenues of biocompatible drug discovery. Metabolites.

[14] Mahtab T (2021). Antibiotic resistance in microbes: History, mechanisms, therapeutic strategies and future prospects. J. Infect. Public Health.

[15] Alhazmi AS, Syame SM, Mohamed WS, Hakim AS (2022). Incorporation of plant extracted hydroxyapatite and chitosan nanoparticles on the surface of orthodontic micro-implants: An in-vitro antibacterial study. Microorganisms.

[16] Shkodenko L, Kassirov I, Koshel E (2020). Metal oxide nanoparticles against bacterial biofilms: Perspectives and limitations. Microorganisms.

[17] Sharma B (2022). Antimicrobial agents based on metal complexes: Present situation and future prospects. Int. J. Biomater..

[18] Claudel M, Schwarte JV, Fromm KM (2020). New antimicrobial strategies based on metal complexes. Chemistry.

[19] Dongre S, Bhagat P (2016). Antimicrobial studies of Rasaushadhies (Herbomineral preparation): A review. Int. J. Ayurvedic Pharm. Chem..

[20] Eshan M, Patil S, Nandi M (2022). An appraisal on antimicrobial activity of herbomineral formulations. Int. J. Ayurvedic Med..

[21] Savarimuthu Michael J, Ranjit Singh AJA, Padmalatha C (2011). Antibacterial potential of some herbo-mineral siddha preparation: An alternative medicine for enteric pathogens. J. Chem. Pharm. Res..

[22] Mahalakshmi G, Raja TS, Pushkala VP (2014). Quality assessment of a traditional siddha drug “ Mupoora Chendurum ”. Am. J. Phytomed. Clin. Ther..

[23] Al-Ansari MM, RanjitSingh AJA, Al-Khattaf FS, Michael JS (2021). Nano-formulation of herbo-mineral alternative medicine from linga chenduram and evaluation of antiviral efficacy. Saudi J. Biol. Sci..

[24] Pooranagangadevi N, Padmapriyadarsini C (2022). Treatment of tuberculosis and the drug interactions associated with HIV-TB co-infection treatment. Front. Trop. Dis..

[25] Bruchfeld J, Correia-Neves M, Kallenius G (2015). Tuberculosis and HIV co-infection. Cold Spring Harb. Perspect. Med..

[26] Yufanyi DM, Abbo HS, Titinchi SJJ, Neville T (2020). Platinum(II) and Ruthenium(II) complexes in medicine: Antimycobacterial and anti-HIV activities. Coord. Chem. Rev..

[27] Nareetsile F, Ndlovu S, Matshwele JTP, Ngaski M (2020). Transition metal complexes with HIV/AIDS inhibitory properties. Chem. Rev. Lett..

[28] Rodrigo C, Fernando SD, Rajapakse S (2020). Clinical evidence for repurposing chloroquine and hydroxychloroquine as antiviral agents: A systemic review. Clin. Microbiol. Infect..

[29] NorRashid N, Yusof R, Rothan HA (2020). Antiviral and virucidal activities of sulphated polysaccharides against Japanese encephalitis virus. Trop. Biomed..

[30] Haraguchi Y, Sakurai H, Hussain S, Anner BM, Hoshino H (1999). Inhibition of HIV-1 infection by zinc group metal compounds. Antiviral Res..

[31] Siddha Formulary of India. (1992).

[32] Thiyagarajan R (2006). Gunapadam, Thathu Jeeva Vaguppu.

[33] Anandhan A, Sakunthala P (2008). Sarakku Suthi Sei Muraikal.

[34] Malarvizhi K, VedhaHari B, Rajalakshmi P, Devaraj S, Ramyadevi D (2023). Analytical insights into the detoxification process and characterization of a traditional metallopharmaceutical formulation. RSC Med. Chem..

[35] Balkrishna A, Chauhan M, Dabas A, Arya V (2022). A comprehensive insight into the phytochemical, pharmacological potential, and traditional medicinal uses of *Albizia lebbeck* (L.). Benth..

[36] Valle-González ER, Jackman JA, Yoon BK, Mokrzecka N (2020). pH-dependent antibacterial activity of glycolic acid: Implications for anti-acne formulations. Sci. Rep..

[37] Alba-romero JDJ (2011). Application of the Alamar blue assay to determine the susceptibility to anti-tuberculosis pharmaceuticals. Afr. J. Microbiol. Res..

[38] Bunalema, L., Tabuti, J., Sekagya, Y., Ogwang, S. & Waako, P. Anti-tubercular activity of *Callistemon citrinus* and *Piptadenistrum africanum* on resistant strains of *Mycobacterium tuberculosis *using Microplate alamar blue assay Anti-tubercular activity of *Callistemon citrinus* and *Piptadenistrum africanum* on resistant (2017).10.5455/spatula.20160316042034.

[39] Priya Dharshini K (2021). pH-sensitive chitosan nanoparticles loaded with dolutegravir as milk and food admixture for paediatric anti-HIV therapy. Carbohydr. Polym..

[40] Barbosa AA, Júnior SA, Mendes RL, de Lima RS, de Vasconcelos Ferraz A (2020). Multifunctional hydroxyapatite with potential for application in theranostic nanomedicine. Mater. Sci. Eng. C.

[41] Sarveswari HB, Kalimuthu S, Shanmugam K, Neelakantan P, Solomon AP (2020). Exploration of anti-infectives from mangrove-derived *Micromonospora* sp. RMA46 to combat vibrio cholerae pathogenesis. Front. Microbiol..

[42] Qa’dan F (2005). The antimicrobial activities of *Psidium guajava* and Juglans regia leaf extracts to acne-developing organisms. Am. J. Chin. Med..

[43] Kannan N, Shanmuga Sundar S, Balaji S, Anil Kumar N, Balasubramanian N (2021). Physiochemical characterization and toxicity assessment of colloidal mercuric formulation—‘Sivanar amirtham’. Colloids Surf. B Biointerfaces.

[44] Kamath SU (2014). Role of purifying agents in chemical transformation of sulphur: An ayurvedic perspective. Asian J. Chem..

[45] Khedekar S, Bedarkar P, Prajapati P (2016). Physicochemical characterization of Shadguna Balijarita Makaradhwaja: A preliminary study. AYU (An Int. Q. J. Res. Ayurveda).

[46] Safardoust-Hojaghan H, Shakouri-Arani M, Niasari MS (2016). Structural and spectroscopic characterization of HgS nanoparticles prepared via simple microwave approach in presence of novel sulfuring agent. Trans. Nonferrous Met. Soc. China (English Ed.).

[47] Sharma V, Samal AK, Pandey S, Chaudhary AK, Srivastava RK (2018). Characterization of Hg-based ayurvedic drug Kajjali: Classical and contemporary approaches. Curr. Sci..

[48] Panda AK, Hazra J (2012). Arsenical compounds in ayurveda medicine : A prospective analysis. Int. J. Res. Ayurveda Pharm..

[49] Adhimeena A, Indrakumar J, Gnanasundari R, Madhavan R (2019). Comparative elemental analysis of siddha Raw drug Thalagam (*Arsenic trisulphide*) by atomic absorption spectrometry technique. Int. J. Adv. Res. Biol. Sci..

[50] Duggal H, Singh G, Kapil A, Mehta D, Kumar S (2022). Elemental and chemical phase analyses of ras-family ayurvedic medicinal products. Biol. Trace Elem. Res..

[51] Song R (1997). Anti-HIV activities of anionic metalloporphyrins and related compounds. Antivir. Chem. Chemother..

[52] De Clercq E (1997). Antiviral metal complexes. Met. Based Drugs.

[53] Sharma R, Bhatt A, Thakur M (2016). Physicochemical characterization and antibacterial activity of Rajata Bhasma and silver nanoparticle. AYU (An Int. Q. J. Res. Ayurveda).

[54] Srinivasulu B, Bhadra Dev P, Murthy PHC (2012). X-ray diffraction analysis of Samaguna balijarita Kajjali (black sulphide of mercury). Int. J. Res. Ayurveda Pharm..

[55] Joshi N (2021). Physico-chemical characterization of kajjali, black sulphide of mercury, with respect to the role of sulfur in its formation and structure. J. Ayurveda Integr. Med..

[56] Wijenayake AU, Abayasekara CL, Pitawala HMTGA, Bandara BMR (2016). Antimicrobial potential of two traditional herbometallic drugs against certain pathogenic microbial species. BMC Complement. Altern. Med..

[57] Kantak S, Rajurkar N, Adhyapak P (2020). Synthesis and characterization of Abhraka (mica) bhasma by two different methods. J. Ayurveda Integr. Med..

[58] Wijenayake A, Abayasekara C, Pitawala A, Bandara BMR (2022). Antimicrobial potential of four mica drugs and their chemical and mineralogical properties. BMC Complement. Med. Ther..

[59] Lekshmi CS, Vineeth PK, Ramesh NV, Lekshmi CS (2020). A proposal for development of biological safety profile protocol for incinerated organometallic preparations (*Ayurvedic bhasmas*). Mater. Today Proc..

[60] Tambekar DH, Dahikar SB (2010). Screening antibacterial activity of some Bhasma (metal-based herbal medicines) against enteric pathogens. Recent Res. Sci. Technol..

[61] Belge R, Pandey R, Itankar P (2018). Synthesis, characterization and antimicrobial study of Vanga bhasma prepared with special reference to Rasatarangini. Int. J. Ayurveda Pharma Res..

[62] Kaur DC, Chate SS (2015). Study of antibiotic resistance pattern in methicillin resistant staphylococcus aureus with special reference to newer antibiotic. J. Glob. Infect. Dis..

[63] Foster TJ (2017). Antibiotic resistance in *Staphylococcus aureus*. Current status and future prospects. FEMS Microbiol. Rev..

[64] Vestergaard M, Frees D, Ingmer H (2019). Antibiotic resistance and the MRSA problem. Microbiol. Spectr..

[65] Sarker MR, Islam KN, Huri HZ (2014). Studies of the impact of occupational exposure of pharmaceutical workers on the development of antimicrobial drug resistance studies of the impact of occupational exposure of pharmaceutical workers on the development of antimicrobial drug resistance. J. Occup. Health.

[66] Elena S (2020). Metal-based nanoparticles as antimicrobial agents: An overview. Nanomaterials.

[67] Karthi, S., Visweswaran, S., Mariappan, A. & Meenakumari, R. Anti-bacterial activity of herbomineral nano preparation padigara parpam, **9**, 1397–1401 (2020).

[68] Khabade S, Rathi BJ, Rathi R, Gupta R (2021). Pharmaceutico-analytical profile of Mrityunjay Rasa and evaluation of its antibacterial activity. Int. J. Ayurvedic Med..

[69] Sharmila CM, Devi RC, Sureka A, Kumar NM, Banumathi V (2018). Evaluation of in-vitro antimicrobial activity of thazhampoo mathirai—a siddha herbomineral formulation. Eur. J. Biomed. Pharm. Sci..

[70] Christian GJ, Ramaswamy RS, Sivaraman D, Nijavizhi M, Anusha S (2016). Anti microbial screening of Siddha Herbo-mineral medicinal formulation Kanagalinga Mezhugu against selected urogenital and enteric pathogens. IOSR J. Dent. Med. Sci..

[71] Tyagi A, Khan AM, Mishra N (2022). Structural, morphological characterization and antibacterial activity of Mahavat Vidhwansan Rasa. Indian J. Nat. Sci..

[72] Shamprasad BR, Lotha R, Nagarajan S (2022). Metal nanoparticles functionalized with nutraceutical Kaempferitrin from edible *Crotalaria juncea*, exert potent antimicrobial and antibiofilm effects against Methicillin—resistant *Staphylococcus aureus*. Sci. Rep..

[73] Meza-villezcas, A. & Gallego-Herna, A. L. Effect of antimicrobial nanocomposites on *Vibrio cholerae* lifestyles: Pellicle biofilm , planktonic and surface-attached biofilm 1–18 (2019). 10.1371/journal.pone.0217869 PMC6561565 31188854

[74] Salem W (2015). Antibacterial activity of silver and zinc nanoparticles against Vibrio cholerae and enterotoxic *Escherichia coli*. Int. J. Med. Microbiol..

[75] Alfuraydi AA, Devanesan S, Al-Ansari M, AlSalhi MS, Ranjitsingh AJ (2019). Eco-friendly green synthesis of silver nanoparticles from the sesame oil cake and its potential anticancer and antimicrobial activities. J. Photochem. Photobiol. B Biol..

[76] Ravichandran M, Aiken GR, Reddy MM, Ryan JN (1998). Enhanced dissolution of cinnabar (mercuric sulfide) by aquatic humic substances. Environ. Sci. Technol..

[77] Kannan N (2018). Physiochemical characterization and cytotoxicity evaluation of mercury-based formulation for the development of anticancer therapeuticals. PLoS One.

[78] Habeeb F (2007). Screening methods used to determine the anti-microbial properties of Aloe vera inner gel. Methods.

[79] Pakadang SR, Hilaria M, Dewi STR, Sinala S, Jumain X (2021). MIC and MKC analysis of herbal medicine in Indonesia against mycobacterium tuberculosis. Pharmacogn. J..

[80] Hochvaldová L, Ve R, Kolá M, Prucek R, Kvítek L (2022). Antibacterial nanomaterials: Upcoming hope to overcome antibiotic resistance crisis. Nanotechnology.

[81] Savarino A, Lusic M, Mai A, Palamara AT, Garaci E (2016). Treatment of latent HIV-1 infections using auranofin or arsenic trioxide. Eur. Patent Spec..

[82] ASTM F 756-13 (2000). Standard practice for assessment of hemolytic properties of materials. Am. Soc. Test. Mater..

