# Supplementary material for: Mercuric-sulphide based metallopharmaceutical formulation as an alternative therapeutic to combat viral and multidrug-resistant (MDR) bacterial infections
Source: Sci Rep. 2023 Oct 4;13:16706. doi: 10.1038/s41598-023-43103-z (PMC10550948; doi:10.1038/s41598-023-43103-z)
Supplement: Supplementary file 1 — Supplementary Information. [file 41598_2023_43103_MOESM1_ESM.docx]

**Supplementary Information**

**Mercuric-Sulphide Based Metallopharmaceutical Formulation as an Alternative Therapeutic to Combat Viral and Multidrug-Resistant Bacterial Infections**

**Malarvizhi Kootharasan,^1^ Ramyadevi Durai^1*,^ Vedha Hari B Narayanan,^1,2*^ Hema Bhagavathi Sarveswari,^3^ Adline Princy Solomon,^3^ Fang H^4^, Luo RH^4^, Zheng YT^4^**

^1^Pharmaceutical Technology Laboratory, School of Chemical & Biotechnology, SASTRA Deemed University, Thanjavur-613401, India.

^2^Centre of Molecular and Macromolecular Studies, Polish Academy of Sciences, Sienkiewicza 112, 90-363 Lodz, Poland.

^3^Quorum Sensing Laboratory, Centre for Research in Infectious Diseases, School of Chemical & Biotechnology, SASTRA Deemed to be University, Thanjavur-613401, India.

^4^Kunming Institute of Zoology, Chinese Academy of Sciences, Kunming, Yunnan, China

*Email: [ramya@scbt.sastra.edu](mailto:ramya@scbt.sastra.edu) / [vedhahari@scbt.sastra.edu](mailto:vedhahari@scbt.sastra.edu)

**Table S1.** Antibacterial activity of Metallopharmaceutical *Sivanar Amirtham* by
Agar well diffusion assay.

| **Organism** | **Zone of Inhibition (mm)** | | | | | |
| --- | --- | --- | --- | --- | --- | --- |
|  | **2.5 mg/mL** | **5 mg/mL** | **10 mg/mL** | **15 mg/mL** | **20 mg/mL** | **25 mg/mL** |
| *S. aureus* ATCC 29213 (MSSA) | - | - | - | 12.3+0.6 | 13.3 ±1.5 | 13.3 ±1.2 |
| *S. aureus* ATCC 43300 (MRSA) | 13.3 ±1.2 | 15.7±2.5 | 18±2 | 18±1 | 20.3+1.5 | 20.7±1.2 |
| *E. faecalis* 29212 | - | - | - | - | - | - |
| *P. aeruginosa*  PA14 | - | - | - | - | - | - |
| *V. cholerae* MTCC 3905 | 21.3±0.6 | 24.0 ±2.0 | 26.7+1.2 | 30.7 ±0.6 | 33.7±0.6 | 34.3±0.6 |


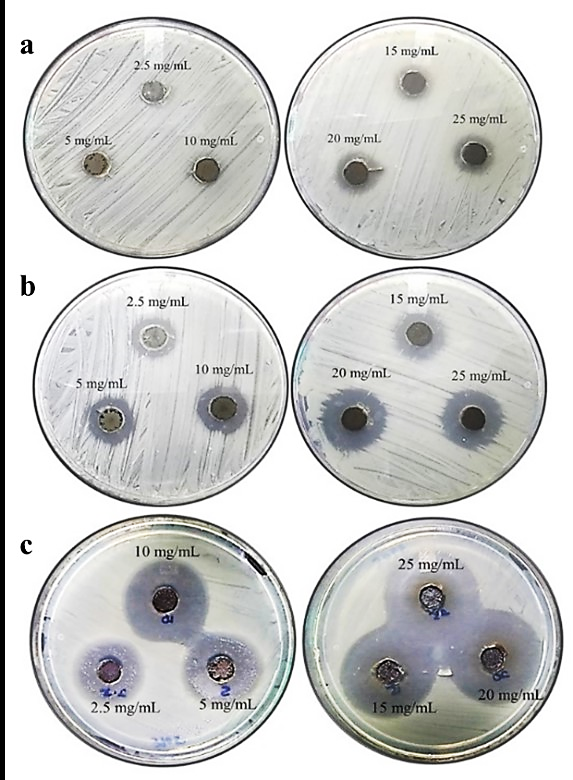


**Figure S1.** Zone of inhibition showing antibacterial activity against pathogens (a) *S. aureus* (MSSA)*,* (b) MRSA and (c) *V. cholerae.*
